# Lamina-associated domains: peripheral matters and internal affairs

**DOI:** 10.1186/s13059-020-02003-5

**Published:** 2020-04-02

**Authors:** Nolwenn Briand, Philippe Collas

**Affiliations:** 1 Department of Molecular Medicine, Faculty of Medicine, University of Oslo, PO Box 1112 Blindern, 0317 Oslo, Norway; 2 Department of Immunology and Transfusion Medicine, Oslo University Hospital, 0424 Oslo, Norway

**Keywords:** 3D genome, Chromatin, LAD, Lamin A mutation, Nuclear envelope, Nuclear lamin, Radial positioning

## Abstract

At the nuclear periphery, associations of chromatin with the nuclear lamina through lamina-associated domains (LADs) aid functional organization of the genome. We review the organization of LADs and provide evidence of LAD heterogeneity from cell ensemble and single-cell data. LADs are typically repressive environments in the genome; nonetheless, we discuss findings of lamin interactions with regulatory elements of active genes, and the role lamins may play in genome regulation. We address the relationship between LADs and other genome organizers, and the involvement of LADs in laminopathies. The current data lay the basis for future studies on the significance of lamin-chromatin interactions in health and disease.

## Introduction

### A snapshot of the genome in three dimensions

In the eukaryotic nucleus, individual chromosomes occupy non-random territories and a radial (i.e., center-to-periphery) disposition enabled by sequestering specific chromosomal domains to the nuclear envelope. Current views of the three-dimensional (3D) genome depict a hierarchical architecture of chromatin [1, 2]. Chromosome conformation capture combined with high-throughput sequencing (a method called Hi-C [3]) makes it possible to map interactions within and between chromosomes across genomes [4]. Hi-C data reveal, on a multi-megabase (mb) scale, a segregation of the genome into open and active “A” compartments, and more closed and inactive “B” compartments. Within compartments, topologically associated domains (TADs) represent regions with a high frequency of chromosomal interactions [5]. Whereas TAD boundaries are overall well conserved, contacts within TADs can vary, partly specifying regulatory contacts. Linearly non-contiguous TADs can also contact each other, defining long-range interactions that can vary between cell types and during differentiation [6–8]. Spatial genome conformation is also modulated by interactions of chromosomes with landmarks such as nucleoli [9], splicing speckles [10], and the nuclear envelope [11], all providing tethering points for chromatin.

### The nuclear envelope as a genome organizer

**Fig. 1:**
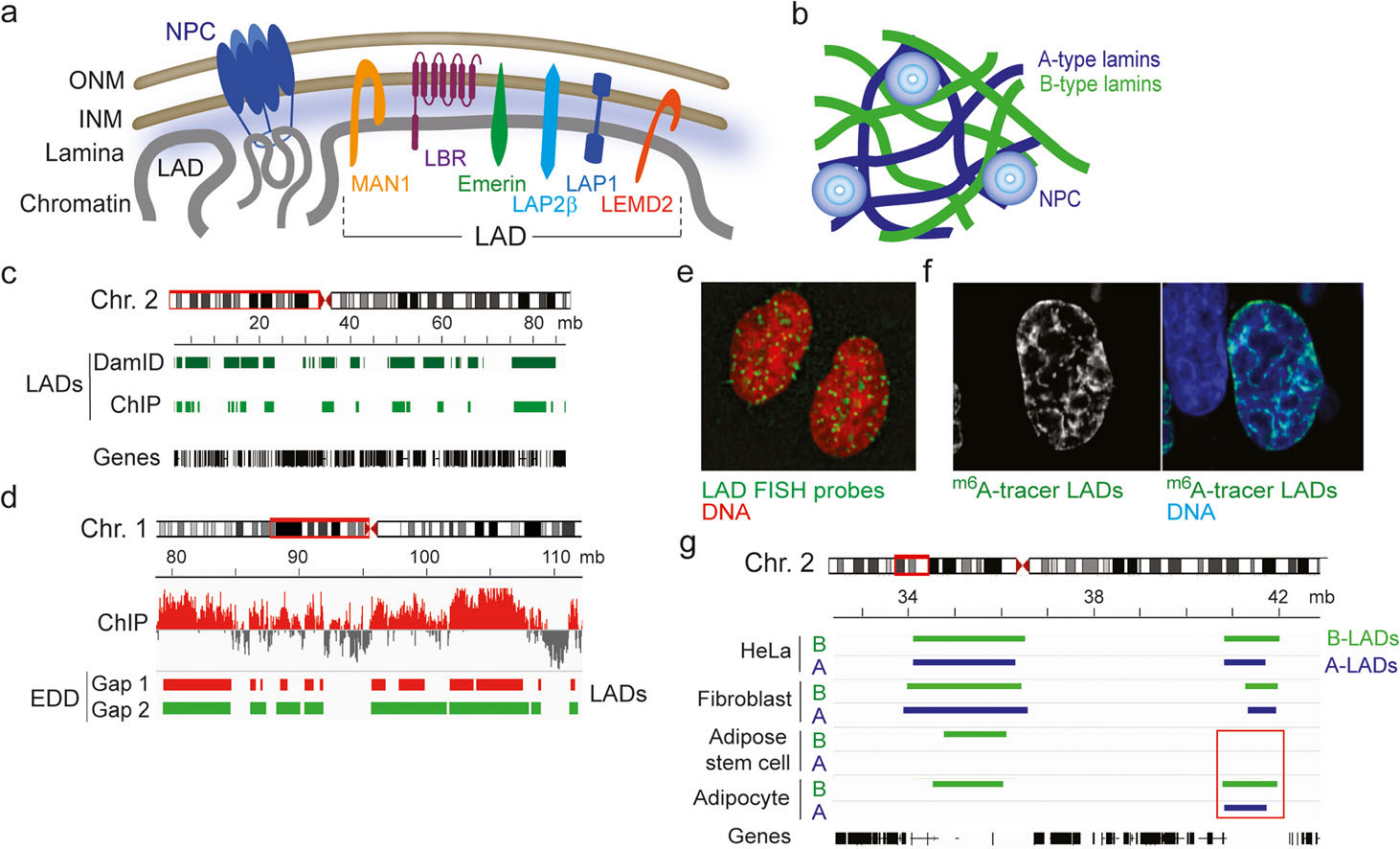
Associations of chromatin with the nuclear envelope. **a** Association of chromatin with the nuclear envelope via inner nuclear membrane proteins, lamina interactions, and interactions with proteins of the NPC. A sample of INM proteins interacting with the nuclear lamina and chromatin is depicted. Chromatin interacting with the NPC is loose and euchromatic, in contrast to the compact and heterochromatic nature of domains interacting with the lamina and INM proteins (LADs). **b** A- and B-type lamins form distinct filaments in the nuclear lamina. **c** Browser view of LADs identified by lamin B DamID-seq and lamin B ChIP-seq in a region of human chromosome 2. DamID data (HT1080 cells) are from [13]; ChIP data (dermal fibroblasts) are from [14]. **d** Detection of LAD using the Enriched Domain Detector (EDD) algorithm is using a tunable gap penalty parameter (Gap). “Gap 1” (here, an arbitrary calling corresponding to EDD’s default gap penalty with this dataset) is more stringent than “Gap 2.” ChIP data are from [14]. **e** FISH visualization of 25 LADs in two nuclei: note the dispersion of LADs around the nuclear periphery and in the nucleoplasm. Reproduced from [15] with permission. **f**^m6^A tracer visualization of LADs. The ^m6^A tracer approach is a DamID variation enabling GFP labeling of Dam-methylated lamin-interacting sites in living cells [15]. Reproduced from [15] with permission. **g** Lamin A LAD (A-LAD) and lamin B LAD (B-LAD) detection by ChIP-seq analysis of lamin A/C and lamin B1 using the EDD algorithm [16], in a region of human chromosome 2. Boxed area, a variable A/B-LAD absent in adipose stem cells but detectable in adipocytes. LAD data are compiled from [8, 14, 16, 17]

At the nuclear periphery, multiple associations of chromatin with the nuclear envelope generate a radial topology of the genome enabling a spatio-temporal regulation of replication and transcription [11]. The nuclear envelope consists of an outer nuclear membrane (ONM) continuous with the endoplasmic reticulum, an inner nuclear membrane (INM) facing the nucleoplasm, nuclear pore complexes (NPCs) and subjacent to the INM, the nuclear lamina, and a meshwork of filament proteins called A- and B-type lamins [12] (Fig. 1a). Radial genome conformation involves interactions of active genome regions with NPCs and of overall silent parts of the genome with the INM and with the nuclear lamina through lamina-associated domains (LADs) [18].

LADs emerge as genomic elements that are both shaped by, and shape, epigenomic states and high-order genome architecture. Mutations in nuclear lamins causing disease can disrupt this organization [19]. Lamin mutations often lead to nuclear envelope deformations and disorganization of peripheral heterochromatin [20], phenotypes also seen in cancer [21, 22] and during normal and pathological aging [23–27]. Here, we highlight current knowledge on the composition and stochastic nature of LADs. We discuss evidence of domain-type and punctual lamin-chromatin interactions at the nuclear periphery and in the nuclear interior, and on how these influence 3D genome architecture and gene expression. We assess how disease-causing mutations in nuclear lamins affect LADs, and provide perspectives on future work expected to shed new light on the roles of lamin-chromatin interactions in health and disease.

## Architecture of the nuclear lamina

### The nuclear lamina is a heterogeneous meshwork of intermediate filaments

The nuclear lamina consists of a meshwork of type V intermediate filaments, the nuclear lamins, and associated proteins [28]. It interfaces and interacts with the INM and chromatin, tethering heterochromatin to the nuclear periphery (Fig. 1a). The main mammalian lamins are lamins A and C (also called lamin A/C), splice variants of the *LMNA* gene, and lamins B1 and B2, products of the *LMNB1* and *LMNB2* genes. Lamins consist of an N-terminal head domain, a coiled-coil central rod domain, and a globular C-terminal tail domain containing an immunoglobulin (Ig)-like fold which in vitro binds nucleosomes [29]. Assembly of the nuclear lamina occurs via polymerization involving homodimerization, head-to-tail assembly of homodimers, and antiparallel assembly of the head-to-tail polymers into filaments [28]. A- and B-type lamins undergo step-wise maturation leading to permanent C-terminal farnesylation and tethering of B-type lamins in the INM, whereas lamin A is further processed C-terminally, untethering it from the INM. Lamin C is never farnesylated, and both lamins A and C are found at the nuclear lamina and in the nucleoplasm where they interact with chromatin [30–32] and regulate chromatin mobility [33]. Lamins A and C also differentially associate with NPC subunits [34] while A- and B-type lamins, by interacting with distinct INM and chromatin components, seem to play distinct roles in nuclear lamina organization [35, 36]. In addition, post-translational modifications of lamins, including phosphorylation [37], *O*-GlcNAcylation (which is specific to lamin A, not C or B) [38], and acetylation [39], contribute to the stability and function of the lamin networks. These observations reflect the complexity of the nuclear lamina and of its role in organizing higher-order chromatin topology.

### The nuclear lamina is a heterogeneous polymer structure

Examination of the nuclear lamina at different scales and resolutions shows that A- and B-type lamins assemble into distinct polymers and form distinct networks (Fig. 1b). Fluorescence resonance energy transfer signals between lamins A and B co-expressed in mammalian cells reveal heterotypic and homotypic interactions between these lamin subtypes in the lamina [40]. Homotypic interactions prevail while heterotypic interactions occur only between A- or B-type lamin homodimers [40]. This suggests that A- and B-type lamins assemble into distinct homopolymers that are able to interact within the lamina. High- and super-resolution microscopy imaging further reveals distinct A- and B-type lamin networks [36, 41] forming distinct “microdomains” [35]. Direct stochastic optical reconstruction microscopy confirms the existence of non-overlapping networks of lamins A and B, and of distinct lamins A and C networks, indicating that lamin subtypes preferentially self-associate (as A-A, C-C, B-B) [34].

Cryo-electron tomography insights into the molecular arrangement of the nuclear lamina reveal a fiber-like morphology of lamin networks decorated with globules, forming filaments [28]. These filaments are packaged into a 14-nm-thick layer (the lamina) but make up only ~ 12% of the lamina volume. Large and variable voids free of lamins are occupied by other proteins and chromatin, emphasizing the structural heterogeneity of the nuclear lamina. Differential labeling of A- and B-type lamins further indicates that their densities and spatial distributions differ, supporting their segregation in the lamina [28]. Additionally, the broad persistence length distribution of lamin filaments—that is, the length under which the lamin polymer behaves as a flexible chain and above which it behaves as a rigid rod—reflects a large degree of flexibility [28] and creates possibilities for A- and B-type lamins to interact with specific protein partners and chromosomal domains.

## Tethering the genome at the nuclear periphery through lamina-associated domains

### Multiple mediators of chromatin interactions with the nuclear envelope

The nuclear periphery is a site of cooperative interactions between the nuclear envelope and chromatin (Fig. 1a). Proteins of the NPC can interact with chromatin, mainly with active regions [11]. INM transmembrane proteins form tissue-specific complexes with lamins and, within these complexes, cooperate to functionally organize chromatin [42–46]. Lamin B receptor (LBR) binds lamin B and the H3K9me3-binding chromobox protein homolog 5 (CBX5) [47], providing a tether for heterochromatin at the nuclear envelope in developing tissues [48]. Thymopoietin/lamina-associated polypeptide (LAP)2β also binds B-type lamins and provides docking sites for chromatin via interactions with histones [49], the DNA-bridging protein barrier-to-autointegration factor [50], and LAP2α [51]. Other INM proteins containing a LEM (LAP2-emerin-MAN1) domain, such as emerin, LEMD2, and LEMD3/MAN1, also bind lamins and chromatin [52].

LAP2β and emerin interact with histone deacetylase HDAC3 [53–55]. HDAC3 contributes to the repressive environment of the nuclear periphery through histone deacetylation [54] and, together with emerin, regulates myogenic gene radial position and expression [56]. Moreover during myogenesis, muscle-specific INM proteins seem to be involved in the radial repositioning of a subset of genes that are downregulated [45]. For example, the *Nid1* gene is repressed in early stages of myogenesis in a manner that correlates with lamina association and a peripheral localization dependent on the INM protein NET39 [45]. Many other genes behave similarly or, in contrast, are downregulated without any lamina association or radial repositioning [45]. Histone methylation is another important feature of the nuclear periphery where histone methyltransferases play a role in sequestering heterochromatin to the nuclear envelope [57–59]. Euchromatin-associated factors and histone acetylation have also been suggested to contribute to the radial segregation of euchromatin and heterochromatin [60]. The multiplicity of chromatin interactions with INM proteins and the lamina, together with the overall heterochromatic nature of the nuclear periphery, contribute to generating concentric “zones” of gene expression with a repressive environment at the nuclear periphery [1, 61, 62], zones of DNA replication timing with late-replicating DNA enriched at the periphery [63], and in some cell types, tissue specificity in sequestering loci to the nuclear envelope [42].

### Mapping LADs using sequencing approaches

The nuclear lamina emerges as an organizer of the radial disposition of chromatin. Evidence of interactions of chromatin with the lamina was not provided until van Steensel and colleagues mapped such associations and coined the term “lamina-associated domain” (LAD) [18] (Fig. 1a). The first report of LADs relied on DamID (Dam identification), a proximity DNA labeling method where the bacterial DNA adenine methyltransferase (Dam) was fused to lamin B1 [18]. Dam is targeted to lamin-binding regions where it methylates adenines at GATC sites to generate 6-methyl-adenine (^m6^A), which does not normally occur in eukaryotes. Regions containing ^m6^A are selectively amplified, and products identified by hybridization to DNA microarrays or high-throughput sequencing (DamID-seq) [13, 18, 64] (Fig. 1c). LADs have also been identified by chromatin immunoprecipitation (ChIP)-seq of lamins A/C or B from chromatin fragmented by micrococcal nuclease digestion or by sonication [16, 27, 32, 65, 66] (Fig. 1c). Despite methodological differences [67], LADs mapped by DamID or ChIP overlap overall [16] (Fig. 1c).

### The bioinformatics factor in LAD identification

How are LADs bioinformatically identified? Accumulating lamin DamID or ChIP sequencing reads to a reference genome is not sufficient to determine areas of enrichment because the overall low level of lamin enrichment on the genome makes it challenging to differentiate occupancy from background. Some lamin ChIP-seq analyses rely on a read count- and threshold-based method in a sliding window [27]. Algorithms detecting domains rather than peaks have however been designed. An algorithm based on the detection of sharp transitions in binarized lamin-genome interactions using a sliding window was used in the pioneering mapping of LADs [18]. DamID data can also be quantile-normalized and segmented by a modified circular binary segmentation algorithm originally designed to detect DNA copy number differences, by testing for significant breakpoints throughout a binned genome [68]; this algorithm has been refined in the LADetector pipeline [69].

LAD detection from “background-adjusted-normalized” lamin ChIP-seq data using a two-state (lamin/no-lamin) hidden Markov model based on the RSEG peak caller has been applied to map changes in lamin B LADs during senescence [65]. RSEG segments the genome by identifying boundaries with high transition probabilities, but ignores the factual enrichment levels [16]. Other domain-calling algorithms such as BroadPeak or PeakRanger do not support the use of input chromatin reads (required to determine enrichment) or only incorporate input reads to validate identified peaks [16].

Given these insufficiencies, we have in 2014 released Enriched Domain Detector (EDD), a domain caller designed to identify LADs from ChIP-seq data [16]. Features of EDD are sensitivity to the width of lamin domains and robustness against background variations [8, 17, 32]. As with any other domain caller, the number of LADs detected with EDD can vary depending on fluctuations in lamin enrichment strength: a local dip in enrichment strength within a LAD can be detected as a break (calling two LADs) or ignored (one LAD) depending on a “gap penalty” setting (Fig. 1d). The definition of a LAD then may come down to a bioinformatics interpretation of a genomic domain enriched in lamins and does not imply a domain where lamin-chromatin interactions are uninterrupted [16, 17, 70].

### Visualizing LADs in the nucleus

While DamID and ChIP are instrumental in identifying LAD sequences, they do not provide information on the localization of LADs in the nucleus. This can be achieved by fluorescence in situ hybridization (FISH) using LAD sequences to design FISH probes (Fig. 1e). FISH can validate DamID- or ChIP-seq data, but the main strength of FISH is in the single-cell resolution of this approach [15, 68, 69].

LADs can also be visualized in living cells. A DamID-derived imaging approach yielding a green fluorescent protein (GFP) ^m6^A labeling of DNA in contact with lamins (an “^m6^A tracer”) allows visualization of DNA sequences that are or have been in contact with the lamina [15]. Lamin-interacting sequences retain the GFP signal even though the interaction no longer exists at the time of observation (Fig. 1f). GFP is fused to a catalytically inactive restriction endonuclease which binds methylated G^m^ATC sites, to visualize binding sites immediately after methylation. The method therefore enables a tracking of LADs in living cells and provides a “molecular contact memory” [15] of lamin interactions with chromatin.

## LADs constitute heterogeneous domains

LADs are detected with various densities on all chromosomes, as domains ranging from 100 kb to 10 mb with a median size of 0.5–1 mb (Fig. 1g). LADs altogether make up to 40% of the genome, but as discussed later, this does not mean that 40% of the genome is bound to the lamina in all cells in a population. LADs overlap with regions of low gene density, typically 2–3 genes per mb (human genome average is ~ 8 genes/mb); replicate in late S phase; and are enriched in B compartments [69, 71]. Accordingly, LADs are rich in di- and trimethylated histone H3 lysine 9 (H3K9me2 and H3K9me3), histone modifications of constitutive heterochromatin, and most genes in LADs are repressed or expressed at low levels [16, 18]. While histone methylation is a mediator of this repressed state [57], histone deacetylation also determines repression in LADs, given the interactions of LAP2β with HDAC3 [54] and of A-type lamins with the sirtuins SIRT1 and SIRT6 [72], which together regulate expression of lamina-associated genes [56]. LADs therefore exhibit typical features of heterochromatin [18].

There are however nuances to this picture. On the one hand, LADs represent intrinsic repressive environments: insertion of reporter genes into LAD sequences is associated with lower expression than in inter-LAD regions [73]. Similarly, sequences determining LAD localization at the lamina inserted into non-LAD regions promote lamina tethering and transcriptional repression [55, 68, 74]. Changes in gene expression concordant with their association with the nuclear lamina have also been shown during differentiation [17, 45, 64, 75].

On the other hand, this relationship is not always straightforward. For example, expression of a subset of T cell-specific genes concurs with detachment of enhancers from the nuclear lamina after T cell activation, while these genes retain a peripheral localization [76]. In other instances, radial repositioning of a lac*O* array in response to alterations in the level of specific INM proteins can occur without any obvious changes in gene expression [43]. This highlights an uncoupling between radial positioning and gene expression. Accordingly, there are also instances of persistent gene expression whether genes interact with or are released from lamins [77]. Further, periodic association and dissociation of genes from the lamina during the circadian cycle in mouse liver are uncoupled from changes in gene expression in LADs or in their vicinity [78]. Nuclear lamina association, therefore, is not the sole determinant of gene repression at the nuclear periphery.

**Fig. 2:**
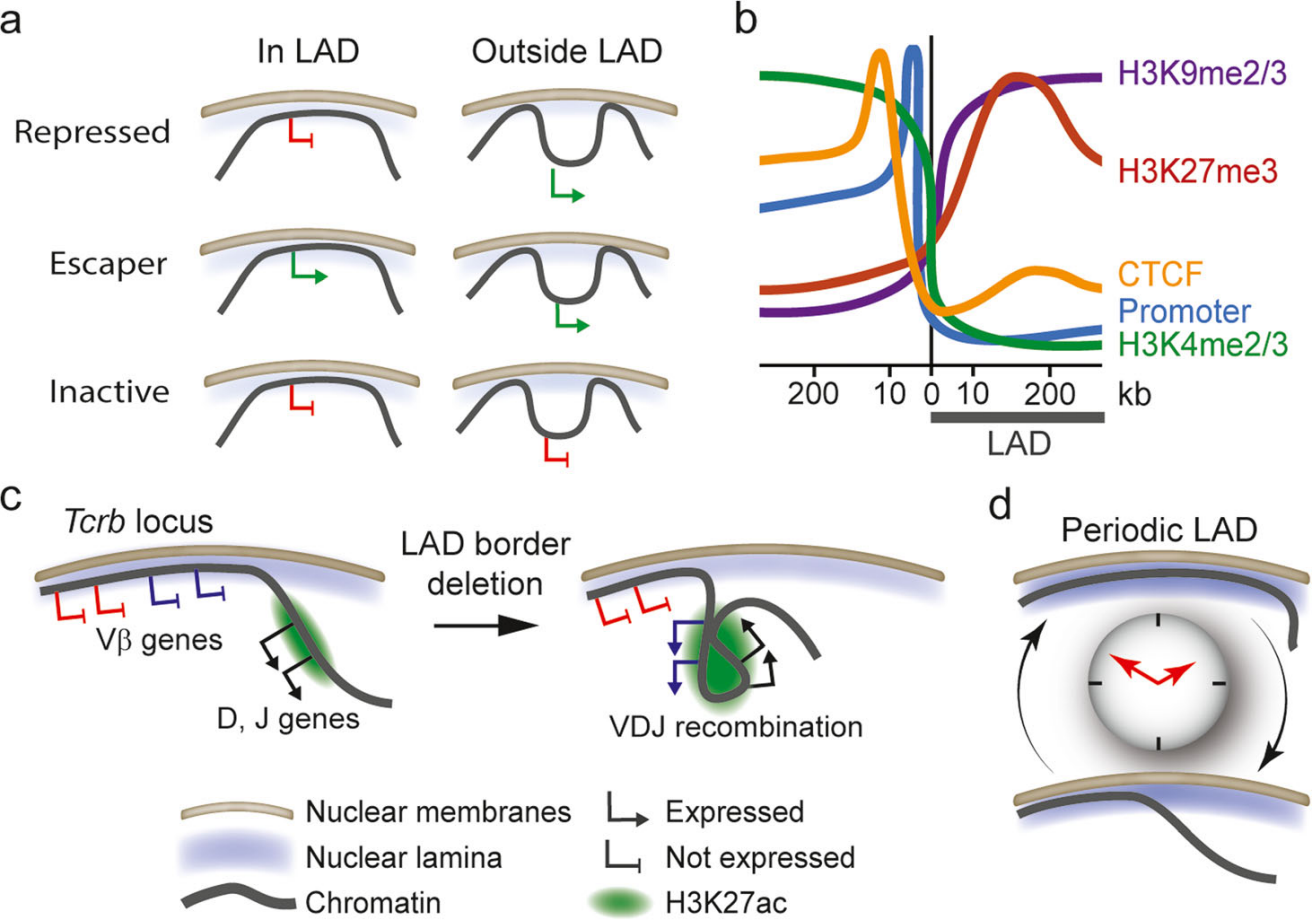
LADs are dynamic and heterogeneous domains. **a** Three classes of promoters identified in LADs: “repressed promoters” become active when experimentally removed from their LAD context, “escaper promoters” are active even when in a LAD, and “inactive promoters” are inactive regardless of their LAD context [80]. **b** Enrichment profiles of indicated genomic features in LAD borders. **c** Deletion of a LAD border within the *Tcrb* locus spatially reorganizes the locus [70]. When inactive, the locus contains a LAD border that separates repressed Vβ genes from the H3K27 acetylated recombination center containing active D and J genes. Deletion of the LAD border elicits spreading of H3K27ac into the LAD, activation of a set of Vβ genes, and looping of the active Vβ genes and the recombination center, favoring VDJ recombination. **d** A subset of LADs in mouse liver displays periodic patterns of association with the nuclear lamina [78]. LAD borders, rather than entire LADs, are most often affected

In fact, at least 10% of genes found at the nuclear lamina are expressed [8, 16, 18, 75, 79]. Using a promoter shuffling strategy where LAD promoters are placed into episomal or endogenous non-LAD contexts [80], three classes of promoters have been identified: “repressed LAD promoters” that become active when removed from their LAD context, “escaper promoters” active even when in a LAD, and “inactive promoters” inactive regardless of their LAD localization (Fig. 2a). So LADs appear to be structurally and functionally heterogeneous, with subdomains, possibly forming micro-loops not bound to lamins [81] escaping the repressive LAD context.

These observations raise the question of what defines a LAD. If a heterochromatic region is sequestered to the lamina (the very definition of a LAD), is being at the lamina necessary to maintain its heterochromatin identity? In the short term, the answer appears to be “no.” However, long-term reduction of lamin B occurring in senescence models leads to a reshaping of heterochromatin and, over time, a shift in LAD organization [27]. This could reflect a more nuanced role for chromatin sequestration at the nuclear lamina, perhaps in helping maintain epigenetic states over time.

## LADs dynamically interact with A- and B-type lamins

### Constitutive and variable LADs

Some LADs occur consistently between cell types and even species, while others are more variable [13]. Constitutive LADs (cLADs) display typical features of heterochromatin, are enriched in repeat elements, and constitute structural elements of the genome tethering chromatin to the nuclear lamina. Variable LAD (vLADs) [13] are a common feature of differentiation and tend to harbor cell type-specific genes that are released from lamins upon lineage commitment [17, 45, 64, 82] (Fig. 1g, red box). This suggests that a fraction of vLADs is developmentally regulated. Most of the variation in vLADs occurs through shifts of LAD borders more frequently than entire LADs appearing and disappearing [64, 68, 77, 83] (Fig. 1g). This suggests that a core LAD structure plays a structural role in nuclear architecture while LAD borders may function in the regulation of expression of LAD-proximal genes [68, 70].

### Lamin A LADs and lamin B LADs

Whereas most lamin A/C LADs (“lamin A LADs”) and lamin B LADs overlap, there are also differences [16, 66, 77] (Fig. 1g) suggesting complementary roles of A- and B-type lamins in genome organization. ChIP-seq analysis of lamins A/C and B from micrococcal nuclease-digested HeLa cell chromatin reveals about one third of LAD genome coverage being unique to lamin B and one fifth unique to lamin A/C [66]. While LADs shared by both lamin types (“lamin A/B” LADs) are likely at the nuclear periphery, lamin A LADs reflect domains not reached by lamin B at the periphery or in the nucleoplasm. Differential interactions of chromatin with A- or B-type lamins at the periphery may be explained by the distinct lamin A- and B-type filaments in the lamina [35] and the dynamic nature of these interactions [77]. Extending these findings, lamin A, A/B, and B LADs can display differential loss or gain of lamin A/C or B interactions, or switch lamin type [8, 17, 77] (Fig. 1g).

What are the consequences of changes in lamin A versus lamin B LAD identity? According to the current data, surprisingly few. LAD reorganization does not correlate with changes in gene expression in LADs [77]. However, 3D genome modeling and FISH data concur in that being in a lamin A LAD or a lamin B LAD differently influences the radial positioning of loci, but this is likely locus-dependent [77]. Radial repositioning of a lamin A LAD also depends on whether a lamin B LAD is nearby [77]. Thus a lamin neighborhood may influence genome topology [33, 84] even though lamins are not directly associated with these regions.

### At the (LAD) border

LADs are typically delineated by abrupt borders. These are characterized by a sharp transition in gene density and gene expression, high promoter and CG dinucleotide density, enrichment in the insulator protein CCCTC-binding factor (CTCF), and abrupt changes in RNA Polymerase II and H3K4me2 [13, 18, 64, 68, 70] (Fig. 2b). Enrichment in H3K27me3 near LAD borders further suggests a role of Polycomb in LAD demarcation to segregate inactive from active genomic segments [68].

The importance of LAD borders has been exemplified at the T cell receptor *Tcrb* locus [70] (Fig. 2c). At the nuclear periphery, a LAD border separates repressed variable Vβ gene segments from the active recombination center containing D and J segments. Deletion of the LAD border leads to spreading of H3K27ac from the recombination center into the proximal LAD portion of the *Tcrb* locus, causing Vβ gene segments to dissociate from the lamina and interact with the recombination center [70]. This is concordant with enhancer activation and increased VDJ recombination [70]. So LAD borders emerge as functionally important structural elements.

### LADs around the clock

Circadian rhythms govern most of our metabolism and physiology [85]. More than 4000 genes exhibit oscillatory expression patterns with defined periodicities in virtually all organs. Circadian gene expression—that is, transcript levels oscillating with a 24-h period—is regulated by circadian patterns of transcription factor binding, histone modifications, and chromosomal interactions [86, 87]. Thus circadian rhythms are implicated in regulating some aspects of 3D genome architecture [88].

Evidence linking the nuclear envelope to circadian gene expression however remains scarce. A handful of circadian genes has been shown in human colon cancer cells to interact with the nuclear lamina in a circadian manner concordant with their repression [89]. In mouse liver, a subset of LAD borders display periodic interactions with lamin B during circadian time [78] (Fig. 2d). This suggests that some LADs may be regulated in a periodic fashion akin to the rhythmicity of chromosomal contacts [87]. Underlying mechanisms remain unknown but could involve periodic binding of lamina-targeting factors. The impact of periodic LADs on gene expression also strikingly remains totally unclear [78]. In fact, clock-control genes lie in an environment deprived of nuclear lamins, permissive to circadian transcription control [88].

### Targeting loci to the nuclear lamina

The sharp transitions in genomic characteristics at LAD borders suggest functionality in genome organization. First, LAD borders seem to harbor sequences important for their radial position. Lamina-associated sequences have been reported as motifs able to target genomic sites to the nuclear periphery [55, 68]. These are enriched in GAGA repeats which constitute binding sites for C-KROX/ZBTB7B, a zinc-finger and BTB/POZ domain-containing protein [55]. ZBTB7B interacts with HDAC3 and LAP2β, suggesting implication of GAGA motifs not only in LAD targeting to the periphery but also in gene repression [55]. Lamina-associated sequences also reveal binding motifs for CTCF and the transcription factor Ying Yang 1 which, perhaps acting as a Polycomb group protein [90], also plays a role in targeting LADs to the nuclear periphery [68].

A FISH-based screen of factors mediating (re)positioning of a set of loci towards the nuclear periphery [91] has identified chromatin modifiers, nuclear envelope proteins, and DNA replication and repair factors as candidates. However, there seems to be no universal factor targeting loci to the periphery: one factor may be implicated in targeting several loci, and one locus may be targeted by more than one factor [91]. Context dependency is probably a key variable, such as cell cycle stage, transcription status, or chromatin state. For example, peripheral targeting of loci has been shown to depend on H3K27me3 at LAD borders [68], G9a-mediated H3K9me2 of sequences flanking LADs or SUV39H-mediated H3K9me3 [92], and in *Caenorhabditis elegans*, step-wise H3K9 methylation [57]. Identification of locus-positioning factors is a critical task in light of pathologies manifested by chromatin reorganization at the nuclear periphery, such as in aging or certain cancers [19, 93, 94].

## How do LADs identified in cell ensembles translate in single cells?

### LAD detection in single cells by DamID

A major advancement in our understanding of LAD behavior is single-cell lamin DamID [15, 95]. A tour de force of nearly 400 single-cell DamID experiments reveals that LADs contacting the nuclear lamina with high frequency are extremely gene-poor, supporting a structural role of cLADs [95]. More variable LADs display less consistency between cells in a population and tend to be specific to a given cell type. Lamin-chromatin contacts may not occur as independent events, but appear to be coordinated intrachromosomally to form LADs in regions found to interact in Hi-C data [95]. This is reminiscent of the formation of intrachromosomal repressive TAD cliques which acquire lamin association and localize at the nuclear periphery [8].

### Not all LADs localize at the nuclear periphery

**Fig. 3:**
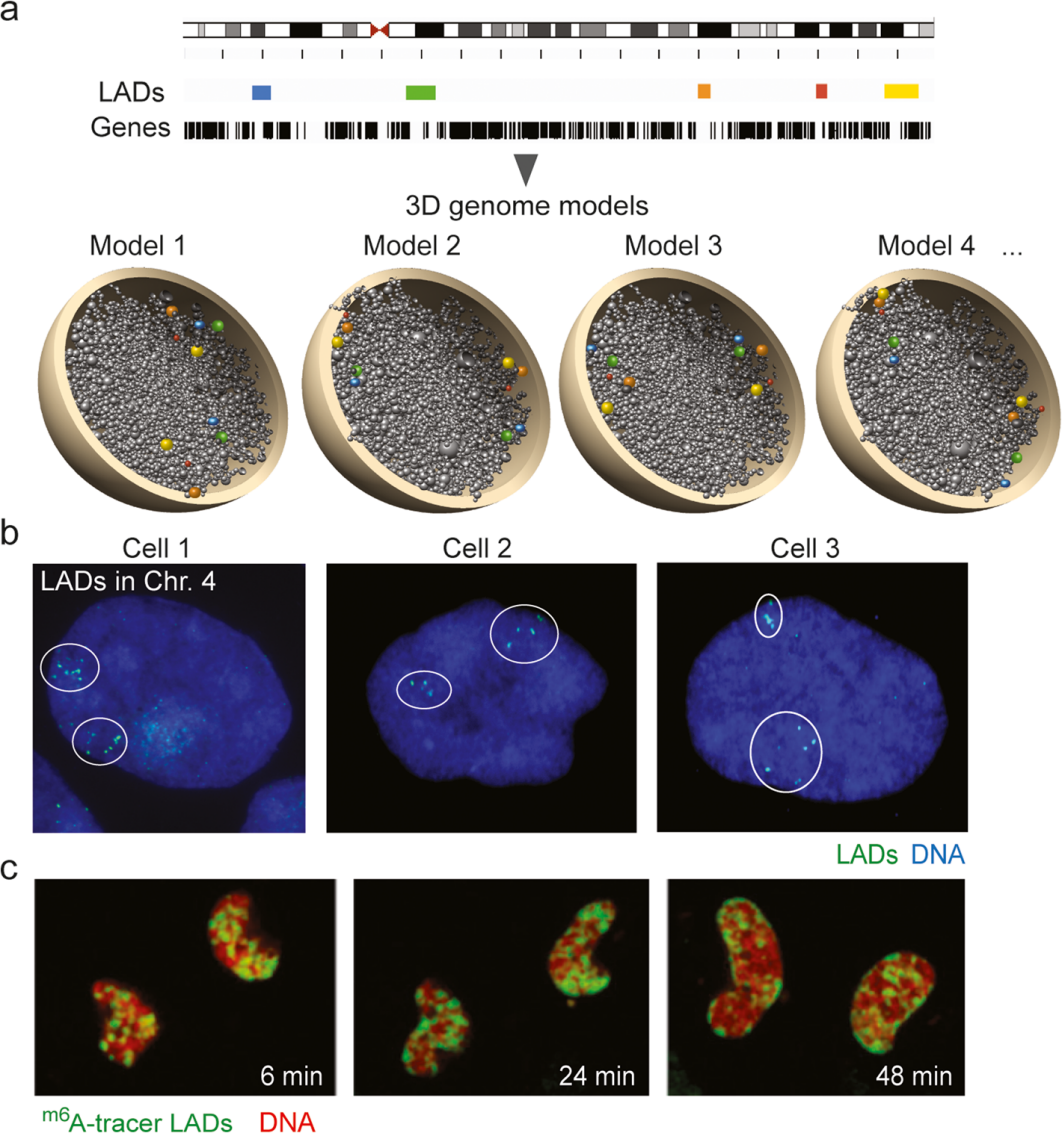
Heterogeneous spatial distribution of LADs in individual cells. **a** Schematic representation of five LADs in a fictive ensemble ChIP-seq experiment (browser view) and in four fictive 3D genome models recapitulating the heterogeneity of LAD positioning in individual cells in a population [14]. **b** FISH image of five labeled LADs (within circles) in both homologous chromosomes 4 in human primary adipose stem cells. Note the variation between cells in the position of these LADs relative to each other and to the nuclear edge, and the heterogeneity of relative LAD positions between two homologous chromosomes (our unpublished data). **c**^m6^A tracer visualization of LAD reassembly post-mitosis in daughter nuclei. LAD targeting to the nuclear periphery occurs during G1 phase [15]. Reproduced from [15] with permission

Imaging of LADs using FISH using probes, and 3D genome structure modeling data, concurs in that only ~ 30% of LADs identified by sequencing map to the nuclear periphery [14, 15, 95]. This makes sense since there is arguably no space at periphery for all LADs found in ensemble methods, which represent up to 40% of the genome [18]. This illustrates the “ensemble *versus* single-cell” problem and heterogeneity of lamin-chromatin interactions between cells [96]. Cell-to-cell heterogeneity in chromatin conformation can be predicted from 3D genome models [14, 97–99] and shown by single-cell Hi-C [100] and by FISH [8, 14, 101] (Fig. 3a, b).

### Single-cell analysis of LAD dynamics

Examination of the 3D organization of LADs in entire chromosomes provides new insights on the role of chromatin-lamina associations in genome organization. FISH-based mapping of LADs and inter-LADs in single chromosomes reveals an arrangement of LADs in constrained domains at the nuclear lamina, which depends on underlying H3K9me2/3 and H3K27me3 and lamin A/C [69]. LADs are largely not stochastic between cells, supporting the structural role of these domains in nuclear architecture [69]. The imaging data also uncover small regions within LADs containing enhancers and TSSs, which escape the repressive environment of the nuclear periphery to interact with A compartment areas; these sites could correspond to escaper promoters [80]. This altogether provides further evidence that LADs constitute discontinuous domains of lamina interaction.

Live-cell tracking of LADs using an ^m6^A tracer in single cells yields information on the dynamics of LADs during nuclear envelope reassembly after mitosis [15, 69] (Fig. 3c). B compartments are established before LAD formation [69]. LAD reformation after mitosis occurs as a step-wise process where LADs in the previous cell cycle display a stochastic distribution in anaphase and telophase [15] (Fig. 3c). During G1, however, LADs relocate to the nuclear periphery [15, 69] (Fig. 3c). The imaging data further suggest that peripheral LADs result from self-aggregation of several small domains in the nuclear interior preceding targeting to the nuclear envelope, with the lamina being resolved in late G1 [69]. This view concurs with the emergence of LADs from intrachromosomal interacting domains [95] in the nuclear interior after mitosis exit [15].

A pending issue in LAD characterization is that of allelic differences. Likely for practical reasons, this has been overlooked in most ensemble ChIP, DamID, or Hi-C analyses, but cannot be ignored in FISH [101] or ^m6^A tracer [15, 69] studies. Positioning of LADs relative to the nuclear periphery even reveals differences between homologous chromosomes [8] (Fig. 3b). Circumventing the issue of ploidy, the single-cell DamID mapping of LADs mentioned above was done in the quasi-haploid KBM7 cell line [95]. A recent allele-specific CRISPR live-cell DNA imaging method allows mapping individual alleles [102] and could be applied to characterize the radial dynamics of LADs.

## Euchromatic LADs

### A-type lamins interact with euchromatin in the nucleoplasm

Even though lamins are mainly found at the nuclear lamina, a pool of A-type lamins exists in the nuclear interior [103–107]. Given the role of A-type lamins in constraining chromatin mobility [33, 108], it is perhaps not surprising that nucleoplasmic A-type lamins interact with chromatin [30] and play a role in gene regulation [109]. What is the evidence?

**Fig. 4:**
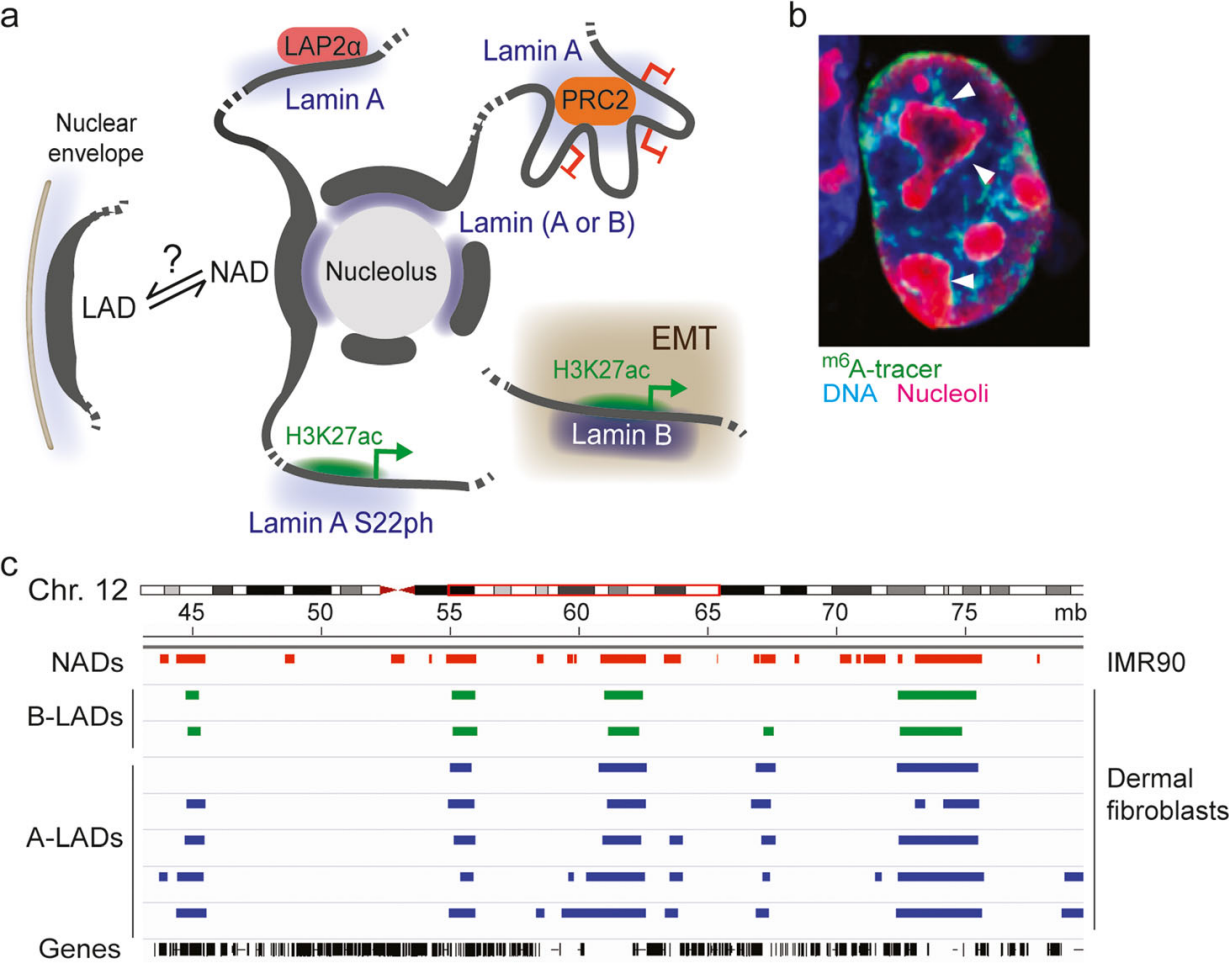
Associations of lamins with chromatin in the nuclear interior. **a** Lamin associations with chromatin in various intranuclear contexts. A-type lamins interact with euchromatic LADs (via LAP2α) [31, 32] and with promoters and enhancers bound by Polycomb (PRC2) [110–112]. Phosphorylated lamin A (lamin A S22ph) also interacts with H3K27ac-rich and active enhancers [113]. Lamins have been shown at the periphery of nucleoli [114, 115], but it remains unclear whether they are implicated in tethering NADs. Lamin B has also been reported to interact with acetylated enhancers and expressed genes during EMT [116] (boxed area). **b**^m6^A tracer visualization of lamin B LADs at the nuclear envelope and in the vicinity of nucleoli. Taken from [15] with permission. **c** Genome browser view of NADs and LADs in a segment of human chromosome 12. Note the overlap between NADs (from [117]) and LADs (from [14])

A-type lamins associate with chromatin in the nucleoplasm in a manner dependent on the chromatin-bound LAP2 variant LAP2α, which directly binds lamin A exclusively in the nuclear interior [31]. There, lamin A interacts with euchromatic LADs overlapping with LAP2α [32] (Fig. 4a). Depletion of LAP2α eliminates the nucleoplasmic pool of lamin A and relocalizes it to the nuclear envelope where it binds heterochromatic domains consistent with typical LADs [32]. Association of lamin A with developmental promoters and enhancers (in the nuclear interior) coinciding with Polycomb repressor complex 2 (PRC2)- and H3K27me3-mediated gene repression has also been shown in pluripotent and multipotent cells [110–112] (Fig. 4a).

Evidence of euchromatic A-LADs also comes from methodological twists. (i) An antibody to lamin A/C which preferentially recognizes its nucleoplasmic fraction immunoprecipitates mostly euchromatin, whereas an antibody preferentially detecting lamin A/C at the nuclear lamina pulls down mostly heterochromatin [32]. (ii) Altering the chromatin sonication regime prior to ChIP can enrich “input” chromatin in euchromatic or heterochromatic regions [32]. This approach reveals a fraction of lamin A/C interacting with euchromatic LADs [32]. (iii) ChIP from chromatin fragmented with micrococcal nuclease also highlights associations of lamin A/C with LADs not typically heterochromatic and containing expressed genes [66]. The variability in chromatin composition and in gene expression patterns in lamin A/C-interacting domains suggests that lamin A/C-chromatin interactions may play different roles in genome organization and gene expression.

### New in the neighborhood: phosphorylated lamin A binds active enhancers

Phosphorylation of A- and B-type lamins regulates their polymerization and nuclear envelope breakdown at mitosis [118]. Lamin A phosphorylation on serine S22 and S392 also occurs in the nucleoplasm in interphase [37], raising the possibility that phosphorylated lamin A could characterize the nucleoplasmic pool of A-type lamins [119]. Lamin A phosphorylated on S22 (lamin A-S22ph) has been reported to bind active enhancers outside LADs [113] (Fig. 4a). In cells from patients with Hutchinson-Gilford Progeria Syndrome (HGPS), a premature aging syndrome caused by a mutation in lamin A [120], ectopic sites gaining lamin A-S22ph are relocalized in the nuclear interior and display increased H3K27ac [113]. These findings suggest new roles of A-type lamins in the regulation of enhancer activity. It will be important to determine whether other instances of association of these lamins with expressed genes [75] can also be explained by their phosphorylated state.

### Euchromatic LADs interacting with B-type lamins

A fraction of B-type lamins has been found to interact with expressed genes, forming narrow euchromatic “e-LADs” during the epithelial-to-mesenchymal transition (EMT) [116] (Fig. 4a). How this might occur is intriguing. Permanent farnesylation of the C-terminus of B-type lamins a priori imposes a lipophilic environment for these lamins, which speculatively could be created by intranuclear lipid microdomains which have been suggested to act as transcription scaffolds [121] and regulators [122]. What lamin B does at active genes containing H3K27 acetylated enhancers [116], how it is targeted there, and whether it coincides with sites of phosphorylated lamin A [113] will be important to address.

## LADs and NADs—where should heterochromatin go?

The nucleolus is the site of ribosome biogenesis where ribosomal DNA genes are grouped and transcribed in a manner dependent on cell growth, metabolism, and differentiation. Roles of the nucleolus also extend to chromatin organization. The nucleolus is a large membraneless organelle that clusters heterochromatin at its periphery in the form of nucleolus-associated domains (NADs) [9, 123] (Fig. 4a). So together with the nuclear lamina, the nucleolar periphery is a site of heterochromatin aggregation [117, 124] (Fig. 4b). NADs have been identified after biochemical isolation of nucleoli and sequencing of the associated DNA [9, 123] (Fig. 4c). NADs are B compartment regions of low gene density and low gene expression and strikingly overlap with LADs (Fig. 4c). NADs therefore display a striking resemblance to LADs, suggesting that the lamina and the nucleolus constitute interchangeable scaffolds for heterochromatin [15, 125] (Fig. 4a, b). NADs could also be LADs accumulating at the nucleolar periphery because lamins also seem to localize there in some cell types [114, 126] (Fig. 4a). What remains unknown is whether these represent distinct lamin fractions and how they might be targeted there, but intranuclear lipid aggregates [121, 122] could perhaps again be implicated.

## LADs and 3D genome topology

### The INM and the nuclear lamina as a spatial nuclear organizer

Association of the genome with INM proteins and the nuclear lamina provides a nuclear periphery perspective of spatial genome organization [11]. LBR plays a key role in tethering heterochromatin at the nuclear periphery in differentiating tissues, whereas this function is taken over by lamin A/C in fully differentiated tissues [48]. However, while lamin A/C or LBR are needed to stabilize peripheral heterochromatin, they are not drivers of heterochromatin assembly, which rather seems to result from a phase separation process involving CBX5 [127]. LBR peptides antagonize CBX5-mediated phase separation [128], in line with the idea that coalescence of heterochromatin can occur independently of nuclear envelope association. Assembly of heterochromatic TAD cliques without LADs [14] further precludes a role of the lamina in the assembly of large heterochromatin domains. This is also in line with the pre-formation of LADs as “lamin-free” intranuclear domains prior to nuclear envelope targeting [69]. However, interactions between heterochromatin and the lamina are necessary for peripheral tethering and to confer a “conventional” organization of chromatin [48, 129]. The importance of LADs in 3D genome topology is also evident from FISH observations that inactive parts of chromosomes interface the nuclear lamina while gene-rich and transcribed regions face the nucleoplasm [69, 130].

Loss of lamins in mouse ES cells causes detachment of peripheral chromatin from the lamina, hence LAD loss [71]. This is accompanied by a decondensation of cLADs, which Hi-C data suggest is mediated by increased inter-TAD interactions and decreased chromosomal contacts within TADs, while TAD boundaries remain unaltered. Chromatin decondensation in cLADs does not reflect a chromosome-wide increase in volume, which contrasts with the impact of lamin B1 knockdown on chromosome decondensation in colon cancer cells [131]. So the impact of the lamina on chromatin organization seems to extend beyond LADs. Further supporting this view, knockdown of lamin A/C increases the number of H3K4me3-marked promoters, yet without necessary changes in gene expression [75]. A lamin A network is also required for proper Polycomb targeting in the genome in myoblasts [110]. Moreover, chromatin compaction in LADs is accompanied by an increase in volume outside LADs in *Drosophila* cells, and depletion of the only *Drosophila* lamin type (Dm0) in these cells (*Drosophila* has two lamin genes, *LamC* and *Dm0*) causes chromatin expansion in LADs [132]. This argues for a role of lamins in shaping the 3D genome also in *Drosophila*.

### Relationship between TADs and LADs

LADs and TADs together emerge as genome organizers in 3D structural models of the genome, at the whole-genome scale [14, 133] and in sub-chromosomal regions [134]. Whole-genome models take into account restraints imposed by TAD-TAD interactions (from Hi-C data) and TAD-lamina interactions (from lamin ChIP-seq data) [14, 133]. Introduction of a LAD constraint on TAD positioning in simulations ascribes TADs towards the nuclear periphery more frequently than if no such constraint is imposed. Similarly to FISH data [95], not all LADs are assigned to the periphery in all genome models [14] (Fig. 3a, b). Evidence for LADs and TADs acting in concert to organize genome topology is supported by Hi-C showing assemblies of TAD cliques during differentiation, translocation of these cliques towards the nuclear periphery (inferred from 3D models), and stabilization at the nuclear lamina [8].

### LADs during embryogenesis: just the beginning

When and how LADs form during development has long remained obscure. Recent DamID maps of chromatin interactions with the nuclear lamina in mouse embryos now shed light on this developmental event [135]. Embryonic nuclear organization is established de novo during the first cell cycles following fertilization. Allele-specific DNA FISH shows that the maternal and paternal genomes establish LADs with distinct features, with maternal LADs being more variable between cells and more euchromatic [135]. LAD formation seems to be independent of DNA replication and may on the paternal genome implicate H3K4 methylation [135]. Embryonic LAD formation seems to precede the detection of topological domains [135], revealing a step-wise assembly of embryonic genome topology.

### LADs as hotspots for de novo mutations

LADs are preferred host domains for de novo DNA lesions, which are under-represented in genes and regulatory regions [136, 137]. LADs are also sensitive to reactive oxygen species and 8-oxoguanine [138]. This raises the question of whether peripheral heterochromatin, rich in repeats [139], would be more susceptible to DNA lesions than the rest of genome, acting as a “sink” for genetic alterations. A more plausible alternative is that DNA lesions are detected more frequently at the nuclear periphery than in the nuclear interior because the compact nature of heterochromatin restricts access to the DNA repair machinery [140, 141]. 3D maps of mutations associated with melanomas also reveal a propensity of the most frequently occurring mutations to be enriched at the nuclear periphery, perhaps for the same reasons [137].

Interestingly, mechanical forces generated by transit of a cell through a micropore seem to preferentially disrupt spatial chromatin organization in B compartments, which contain LADs, over euchromatic A compartments [142]. This could be linked to the relative peripheral localization and rigidity [143] of heterochromatin which could lead to slow recovery from damage. The mechanical properties of heterochromatin could again protect active gene domains from disruption.

### Hand in hand: post-translational modifications of lamins and chromatin

Nuclear lamins can be post-translationally modified by phosphorylation [37], β-*O*-linked *N*-acetylglucosamine (*O*-GlcNAc) modification [38], and acetylation [39]. Lamin A-*O*-GlcNAc [38] predominantly occurs at 11 sites in the C-terminal tail, between residues 385 and 646, which include the nucleosome-binding Ig fold [29]. This bulky nutrient-sensing modification of lamin A may affect nuclear lamina structure and hypothetically interactions of A-type lamins with chromatin. Integral proteins of the INM are also post-translationally modified [144], not only expanding the proteome of the nuclear envelope, but also plausibly modulating associations with the lamina and chromatin.

Lamin A acetylation is essential for maintenance of nuclear architecture and genome integrity [39]. Loss of acetylation results in enhanced lamin solubility, impaired phosphorylation, and mechanostability. It also results in nuclear blebs, a phenotype resulting from disease-causing lamin A mutations [20], and in chromatin anomalies manifested by enrichment in H3K27me3, reduction in H3K27ac and reduced chromatin accessibility [39]. Lamin acetylation could therefore potentially affect interactions with chromatin. Acetylation of lamin A at lysine K311 may be health-relevant: expression of the lamin A acetylation-deficient K311R mutant causing a muscle dystrophy [145] phenocopies chromatin modifications elicited by the loss of the lamin A/C acetylase complex [39], implicating lamin A/C acetylation in laminopathies.

## Implications of LADs in disease

### LADs and cancer: links remain speculative

Cancer cells are characterized by changes in their epigenome akin to changes in gene expression [146]. Alterations in DNA content through genomic duplications and deletions, along with deformations of the nuclear envelope [21], suggest impairments also in associations of the nuclear lamina with chromatin. However, LADs have not systematically been examined to date, despite their mapping in several cancer cell lines [16, 77]. One limiting factor may be the probable variability in LADs between cells within a tumor which could exceed LAD variations between normal diploid cells [94]. So there is to date no evidence of systematic alterations in LADs during cellular transformation, or of any causal link between LADs and cancer [22, 94].

### Laminopathies and LADs

**Fig. 5:**
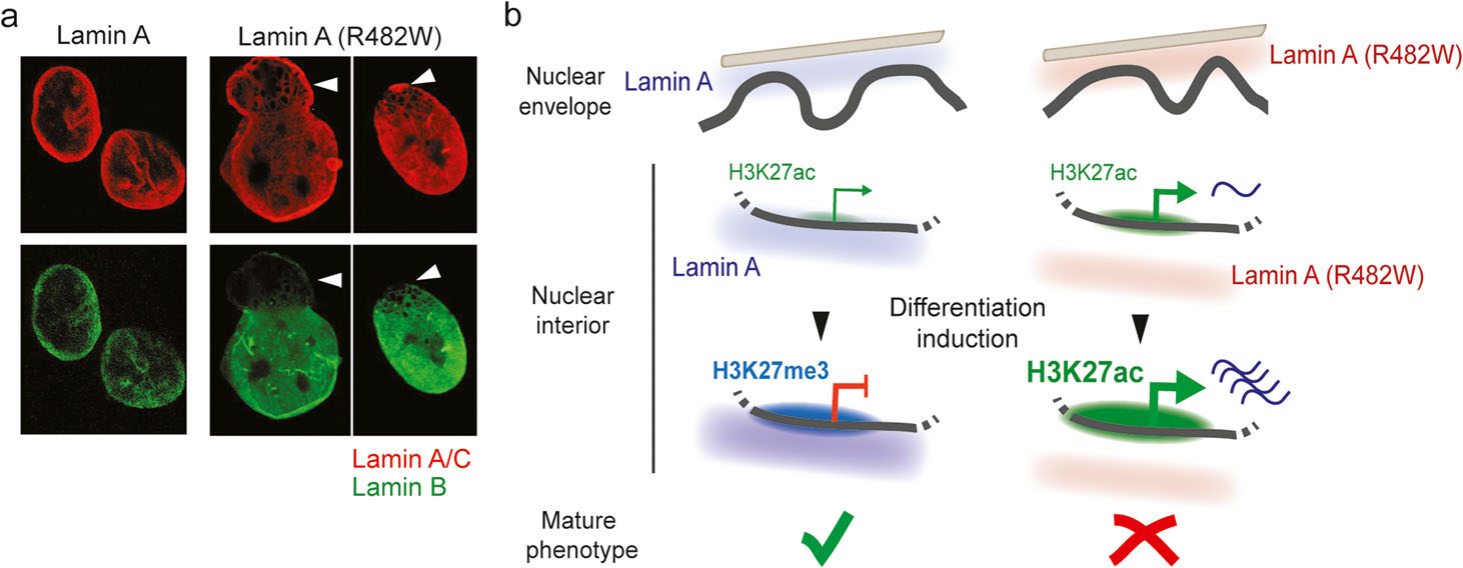
Lamin A mutations causing laminopathies alter peripheral and internal nuclear architecture. **a** Nuclear envelope deformations in the nuclei of patients bearing the FLPD2-causing lamin A (R482W) mutation. Note the nuclear blebs essentially devoid of lamin B (arrows); reproduced from [20] with permission. **b** The lamin A (R482W) mutant elicits chromatin rearrangements at the nuclear periphery and in the nuclear interior in patient cells and in stem cell models of FPLD2 [14, 111, 112]. Whereas peripheral A-LADs are moderately affected at the nuclear periphery, punctual (non-LAD) lamin A interactions with promoters and enhancers in the nuclear interior are impaired in cells expressing lamin A (R482W). In wild-type cells (left), a weakly H3K27-acetylated developmentally regulated promoter bound by lamin A is inactivated during differentiation, in association with increased lamin A binding and H3K27 methylation. In mutant cells (right), defective binding of lamin A (R482W) coincides with expression of the gene in the undifferentiated state, and unscheduled overexpression and H3K27 acetylation after induction of differentiation. Differentiation is, however, abortive [111, 112]

A causal link between mutations in A-type lamins and Emery-Dreifuss muscular dystrophy has been established over two decades ago [147]. Since, ~ 500 mutations throughout the *LMNA* gene have been connected to ~ 15 diseases commonly called laminopathies. These diseases exhibit distinct or partly overlapping phenotypes and include muscle dystrophies, partial lipodystrophies, peripheral neuropathies, and HGPS [19]. Nuclei of cells from laminopathy patients typically show nuclear membrane deformations (Fig. 5a). Several lamin A mutations affect interactions with chromatin, epigenomes, and genome conformation. Expression of lamin A mutants linked to a progeroid syndrome (lamin A [L647R]) [148] or to a congenital lipodystrophy and muscle dystrophy (lamin A [R388P]) [149] results in LAD differences consistent with the peripheral (L647R) or nucleoplasmic (R388P) localization of these mutants [14]. Accordingly, 3D genome models incorporating Hi-C data and LADs generated by expression of these mutants predict distinct LAD radial association densities [14].

More relevant in a laminopathy context is the mapping of LADs in fibroblasts from patients with the lamin A (R482W) mutation causing familial partial lipodystrophy of Dunnigan type (FPLD2). This mutation lies in the Ig-like fold of lamin A and perturbs DNA and nucleosome binding in vitro [29, 150]. Lamin A LADs in FPLD2 fibroblasts and 3D genome modeling reveal patient-specific LADs in the nuclear interior containing genes relevant for adipose tissue differentiation and function [14]. Alterations in chromatin conformation inferred from these findings are another manifestation of large-scale chromatin changes in HGPS, such as the loss of LADs and of heterochromatin at the nuclear periphery [24, 26].

However, whether LAD defects observed in overexpression systems and in cultured patient cells are the primary causes of cellular and organismal pathologies remains questioned because changes in LADs are usually minor [14, 151]. Recent data also indicate that A/B re-compartmentalization elicited by a cardiomyopathy-causing lamin A truncation in an induced pluripotent stem cell model of the disease can hardly be linked to the pathophysiology in this model [152]. Thus, LAD alterations associated with lamin A mutations do not necessarily support a hypothesis of chromatin defects being at the origin of laminopathies.

### Local impact of lamin mutations on promoters and enhancers

On the other hand, during EMT, lamin B1 binds ectopically to the TSS of highly expressed genes [116], and in HGPS cells, lamin A-S22ph is found at enhancers showing elevated H3K27ac [113]. Accordingly, the FPLD2-causing lamin A (R482W) mutation is associated with local defects in chromatin organization (Fig. 5b). In an adipose stem cell model, expression of the mutation results in increased reduced H3K27me3, increased H3K27ac, and increased looping (suggested by FISH) of a distal enhancer onto the anti-adipogenic microRNA *MIR335* gene, concordant with its overexpression and abortive adipogenesis [112]. Defective Polycomb-mediated repression similarly emerges from an induced pluripotent stem cell model of FPLD2 focusing on endothelial differentiation [111], and from ectopic H3K27me3 in myoblasts expressing a muscle dystrophy-causing lamin A mutation [110]. These studies suggest that defective lamin binding could underline pathology phenotypes.

Even though LADs, punctual inter-LAD lamin-chromatin interactions, and chromatin topologies may be altered in models of laminopathies, the chromatin defects reported above might nonetheless be secondary or parallel to structural tissue and cellular perturbations, inferring mechanotransduction perturbations to the nuclear lamina and the genome. Studies will need to disentangle direct gene regulatory effects from impacts of the mutations on the mechanotransduction apparatus.

## Perspectives

Evidence for structural heterogeneity of the nuclear lamina and LADs prompts further investigations on mechanisms regulating local variations in lamin-chromatin interactions within LADs and variable punctual interactions in inter-LADs. The detection of LADs with preferences for A- or B-type lamins warrants investigations on whether these occur at the nuclear lamina and through what mechanism, and whether they are related to the formation of microdomains of lamins A/C and B in the lamina. These observations also raise the question of if and how stoichiometry of A- and B-type lamins, which varies with tissue stiffness [153], influences LADs and radial chromatin distribution. Similarly, to what extent lamina structure and the distribution of lamin A and lamin B LADs are affected by disease-causing lamin A mutations is unknown.

Phosphorylation of lamin A/C provides a hint on the importance of post-translational lamin modifications on chromatin binding and enhancer activity [113] and gene regulation in a more global sense. The binding of lamin B1 on enhancers that are activated during EMT however remains intriguing [116]. Are these interactions regulatory or does lamin binding occur to “seal the deal,” locking a chromatin conformation in place? The lamin A (K311R) prevents lamin A/C acetylation at this residue, which leads to nuclear envelope deformation, lower chromatin accessibility, and histone acetylation defects [39], indicating a direct involvement of A-type lamins in local chromatin composition and gene regulation. Similarly, how lamin A mutations affect lamin modifications, including the nutrient-sensing GlcNAcylation, remains unexplored territory.

Increasing awareness of not only between-cell variations, but also variations between homologous chromosomes in LADs and spatial chromatin arrangement [101], raises the issue of whether there are allele-specific LADs. Single-cell imaging approaches in polymorphic backgrounds should provide insights on chromosome-specific LADs and on whether regulation of imprinted gene expression might also involve lamin interactions.

More remains to be learned on the relationship between mechanical tension and stress, the nuclear lamina, LADs, and genome architecture. Nuclear volume differs between cells cultured in 2D or 3D. Increase of nuclear volume is accompanied by chromatin expansion, unfolding of the nuclear lamina, and increase in nucleolar volume [154]. As such, changes in nuclear size and shape during cell migration may impact gene expression by altering 3D chromatin architecture [142]. What are the consequences of unfolding the nuclear lamina on lamin-chromatin interactions, chromatin compaction in LADs, epigenetic marks, and gene expression in these regions? Are LADs at all maintained and if so, are they structurally altered? A 3D chemo-mechanical model to describe a 3-way feedback between cell adhesion, cytoskeleton, and the nucleus has been proposed [155]. The model argues that tensile stress regulates properties of the nucleus and chromatin, including lamin A/C levels, chromatin stiffness, and histone acetylation. The data lead to the view that the nuclear lamina is a mechanosensing mediator of tensile stress. Should these effects be mediated through LADs, impacts on chromatin architecture and gene expression may be anticipated [156].

Interactions of lamins with the genome turn out to be a lot more subtle and complex than originally described. Domain versus punctual interactions, nuclear periphery versus internal interactions, LAD heterogeneity and dynamics between cells, lamin modifications and disease-causing lamin mutations altering LADs, epigenomes and the 3D conformation of chromatin make the study of LADs as genomic organizers more exciting than ever.

## Supplementary information

**Additional file 1.** Review history.

## Abbreviations

3D: Three-dimensional
ChIP-seq: Chromatin immunoprecipitation sequencing
DamID: Dam identification
FISH: Fluorescence in situ hybridization
FPLD2: Familial Partial Lipodystrophy of Dunnigan Type
GFP: Green fluorescent protein
HGPS: Hutchinson-Gilford Progeria Syndrome
INM: Inner nuclear membrane
LAD: Lamina-associated domain
cLAD: Constitutive LAD
vLAD: Variable LAD
NAD: Nucleolus-associated domain
NPC: Nuclear pore complex
ONM: Outer nuclear membrane
TAD: Topologically associated domain
