# Supplementary material for: Lamina-associated domains: peripheral matters and internal affairs
Source: Genome Biol. 2020 Apr 2;21:85. doi: 10.1186/s13059-020-02003-5 (PMC7114793; doi:10.1186/s13059-020-02003-5)
Supplement: Supplementary file 1 — Additional file 1. Review history. [file 13059_2020_2003_MOESM1_ESM.docx]

Review History

**First round of review**

**Reviewer 1**

**Comments to author:**

Briand and Collas have written an excellent and comprehensive review on lamina-associated domains (LADs). LADs were initially considered to represent the inactive parts of the genome positioned in dense heterochromatin close to the nuclear periphery. However, intensive efforts from many groups have revealed that LADs can be quite dynamic, differ between cell types and even be enriched in active chromatin. The causative link between mutations in nuclear lamins and human diseases provide an obvious biomedical interest in understanding the behaviour and role of LADs but this also extends into fundamental cell biology and development. The Collas group has contributed many important discoveries on LADs and the current manuscript reflects the expertise of the group.

My comments are minor:  

Page 2, line 42.          
Typo: 'he' -> 'the'

Page 2, line 49; Page 10, line 8; Page 17, line 24.
The authors mention changed in heterochromatin (distribution) in normal aging, but what is the evidence? The cited references are reviews and/or paper focusing on disease conditions. The Misteli group published in 2006 (PMID: 16645051) that fibroblasts from healthy old donors have several changes in chromatin marks and HP1 proteins, but perhaps the authors could refer to more evidence?

Page 4, line 44.
The authors write that 'During myogenesis, tissue-specific INM proteins also target myogenic genes to the nuclear lamina where they are repressed [34].' This sounds counterintuitive and I think this is only true for some myogenic genes and not necessarily as a class. Perhaps this should be elaborated a bit, e.g. 'a subset of myogenic genes' [e.g. in ref 34: "The Nid1 gene is normally repressed early during myogenic differentiation"]. Moreover, many non-myogenic genes were affected too in ref 34.

Page 4, line 46.
When the authors discuss the role of (repressive) histone marks on tethering heterochromatin to the nuclear periphery, perhaps it would be relevant to mention that euchromatin-associated factors have recently been proposed to contribute to segregation of heterochromatin, e.g. PMID: 31118512.

Page 5, line 24.
The importance of the particular bioinformatics tools used to identify and define LADs is a very relevant aspect. Given the expertise of the Collas group in this aspect, perhaps the authors could provide a figure to illustrate the differences between the cited methods.

Page 6, lines 48 and 59.
I don't think the citation to ref 61 is correct here. The study by Reddy and coworkers inserted a reporter gene randomly into the genome without knowing (or characterising) if it landed in a LAD or outside.

Page 7, line 38.
The concept of micro-loops is interesting. Here the authors mention this in the context of ref 71, which is quite recent. However, the concept was proposed much earlier. See for instance Figure 9 in PMID: 21176223 and probably elsewhere.

Page 8, line 51.
Typo: 'nearLAD' -> ' near LAD'

Pages 10-11, refs 55 and 59.
I'm a fan of pre-print initiatives like bioRxiv, but I also think that the caution note on the bioRxiv page ('This article is a preprint and has not been certified by peer review') should be taken serious during citation. I haven't looked carefully at the data in refs 55 and 59, nor is it my attention to criticise those manuscripts. However, it strikes me that ref 55 and 59 were posted more that 3 and 2 years ago, respectively, and have not been published in peer-reviewed journals yet. Moreover, comparing the abstracts, a couple of sentences related to the main data of the manuscripts are identical, suggesting that some data from ref 55 were 're-used' again in ref 59. I insist that I'm not saying that the authors should not cite those two pre-preprints, but I suggest them to pay extra attention when citing non-peer-reviewed material.

Page 11, line 15.
The observation that LADs relocate to the nuclear periphery was made in ref 58 - and indeed the figure is from this ref - so would be more appropriate to cite ref 58 instead of ref 59.

Page 12, line 20.
'Variable chromatin composition and gene expression states lamin A/C-interacting domains suggests…'. I don't understand this sentence. Please rephrase.

Page 14, line 40.
Please clarify that although the particular cell line used in ref 122 only expresses Dm0,
Drosophila has indeed two lamin genes, Dm0 and C.

Page 16, line 43.
'These diseases…' Please consider adding a reference to this sentence.

Figures:
-     It seems that Figures 4 and 5 were uploaded in the wrong order.
-     Figure 1 legend: 'LAD data (dermal fibroblasts) are from [84].' should be 'ChIP data (dermal fibroblasts) are from [84].'
-     Figure 1 legend: 'lamin B1using' should be 'lamin B1 using'.
-     Figure 3d: What is the purpose of this panel? The information is already present in panel b.
-     Figure 5: Is panel 5b needed? Same phenotype is depicted (and more clearly demonstrated) in 5a.

**Reviewer 2**

**Comments to author:**

Authors Briand and Collas have provided a timely and thorough review of genome organization through the lens of lamina-associated domains (LADs) and lamin biology. This review describes our current understanding of the role of lamins in positioning lamina-associated domains (LADs) to the nuclear lamina. Overall this review is very timely and provides a needed integration of what is known about lamin biology and LAD organization. I hope that my critiques, summarized below, help to strengthen this very nice review.

Critiques (in order of presentation, not importance)
1) In the abstract there is a sentence that reads "We discuss findings of lamin interactions with regulatory elements of active genes, challenging the dogma of lamins playing a repressive role on transcription." Indeed, in the manuscript the authors offer a much more nuanced view than this sentence would indicate. The majority of data presented in the review supports a major role for LADs being repressive environments, but with some exceptions to that "rule". The sentence in the abstract should reflect this. Please remember this is for a general audience. Something like "While LADs contain mostly repressed genes, we discuss findings of lamin interactions with regulatory elements of active genes, thus expanding our view of the role that lamins may play in genome regulation"

2) The use of the word "anchoring" to describe lamin/LAD interactions is a bit of an issue with me. This suggests a much more solid type of interactions than has been shown by existing data. connotatively this implies a direct and very stable set of interactions. Perhaps better words in lieu of "anchor": associate/association, position/positioning, recruit/recruiting etc.

e.g. in the intro: …a radial disposition enabled by sequestering specific chromosomal domains to the nuclear envelope."

3)in the paragraph beginning "LADs emerge as…" Should be restated: "LADs emerge as genomic elements that are both shaped by and shape epigenomic states and higher-order genome architecture". LADs are not only driving epigenetic and genome organizational changes, they are also shaped by these changes. You provide the support for this altered statement throughout the manuscript.

4)"A-type lamins are further processed C-terminally, untethering them…" Incorrect. Only lamin A is processed in this way, lamin C is never farnesylated.

5)The paragraph beginning "the biochemical properties…" are a bit duplicative. These paragraphs should be combined to provide a more fluid narrative.

6)In the paragraph describing super-resolution studies, it is not mentioned that lamin C also forms a distinct network This is important, especially since you mention the preferential filament interactions at the end of this paragraph. I may have missed it, but the microdomains discussed here are also differentially associated with different chromatin and NE components. E.g. lamin C preferentially interacts with NPC (Burke group-proteomics and super-res), while other filaments interact with different INM/chromatin (eg. Shimi et al, and Burke group). I think there is value to describing that here potentially (or in the mediators section)

7) Zullo et al. also found HDAC3 interacting with Lap2b—this in the context of LAD organization, might be worth mentioning here (this study is mentioned later in the manuscript)

8) "..a gene repression gradient" Is it a gradient? Or do these associations create a "zone" as described inYao et al 2011 (Tijan group) and suggested in a review by Luperchio et al., 2014. Perhaps this is splitting hairs, but a gradient implies throughout the nucleoplasm, whereas a zone implies a delimited gradient.

9)In the paragraph that begins "Accordingly, LADs are rich in .." the Lap2b with HDAC3 should also be mentioned here (can refer back to original description earlier in text).

10) In the paragraph that begins "On the other hand, this relationship.." the authors describe a set of circumstances in which changes in LADs do not correlate with changes in expression.
a) uncoupling between radial positioning and gene expression level (as stated before, the radial positioning argument falls flat for many reasons
b) genes are "bound to or released from A- or B-type lamins"—the word "bound" is too strong. "interact" is more appropriate.
c) this raises the separate question of what defines a LAD. If a heterochromatic region is sequestered to the lamina, is being at the lamina necessary for maintiaing its heterochromatin identity? In the short term, the answer appears to be "no". Over the longer term, this *may not* be true—e.g. as the Berger group has shown, over time there is a shift in LAD organization in senescence models where lamin B1 is depleted. This reflects perhaps a more nuanced role for sequestration: helping maintain epigenetic state over time.

In short, I think that adding this nuance will help this section and will contextualize these findings a bit more.

11) In the paragraph that begins "What are the consequences in changes in LAD identity?" This is confusing. Please change to "What are the consequences in changes in A versus B LAD identity?"

Also, I generally find this nomenclature a bit confusing since A/B compartments are used in HiC data.

12) In the section screening for factors mediating locus targeting: I think it is important to discuss several papers that have implicated chromatin state as important for localization: Kind et al, Harr et al., Bian et al., Towbin et al.. This is needed nuance here and can follow your sentence about context dependency.


13) LADs as hotspots for mutations: Since LADs and the B-compartment overlap significantly, I think it is worthwhile to discuss that mechanical forces generated by transiting a micropore seemed to effect B-compartment chromatin specifically (Jacobson, BMC 2018). This also supports a later point in the manuscript musing on the role of mechanotransduction in LAD organization and regulation.

14) PTMs: INM proteins are also heavily modified. This should be mentioned and referenced in this.

**Authors Response**

**Point-by-point responses to the reviewers’ comments:**

Reviewer #1: Briand and Collas have written an excellent and comprehensive review on lamina-associated domains (LADs). LADs were initially considered to represent the inactive parts of the genome positioned in dense heterochromatin close to the nuclear periphery. However, intensive efforts from many groups have revealed that LADs can be quite dynamic, differ between cell types and even be enriched in active chromatin. The causative link between mutations in nuclear lamins and human diseases provide an obvious biomedical interest in understanding the behaviour and role of LADs but this also extends into fundamental cell biology and development. The Collas group has contributed many important discoveries on LADs and the current manuscript reflects the expertise of the group.

My comments are minor:

Page 2, line 42.

Typo: 'he' -> 'the' - Corrected

Page 2, line 49; Page 10, line 8; Page 17, line 24.

The authors mention changed in heterochromatin (distribution) in normal aging, but what is the evidence? The cited references are reviews and/or paper focusing on disease conditions. The Misteli group published in 2006 (PMID: 16645051) that fibroblasts from healthy old donors have several changes in chromatin marks and HP1 proteins, but perhaps the authors could refer to more evidence?

We have added relevant references to distinguish NE deformations in cancer and in normal and pathological aging.

Page 4, line 44.

The authors write that 'During myogenesis, tissue-specific INM proteins also target myogenic genes to the nuclear lamina where they are repressed [34].' This sounds counterintuitive and I think this is only true for some myogenic genes and not necessarily as a class. Perhaps this should be elaborated a bit, e.g. 'a subset of myogenic genes' [e.g. in ref 34: "The Nid1 gene is normally repressed early during myogenic differentiation"]. Moreover, many non-myogenic genes were affected too in ref 34.

The reviewer highlights several good points. We have revised the text accordingly by adding details and the Nid1 example. As suggested, we have also added a note saying that “…however, many other genes, including non-myogenic genes, behave similarly or are down-regulated without any lamina association or radial repositioning {Robson, 2016}”. The text is now more accurate.

Page 4, line 46.

When the authors discuss the role of (repressive) histone marks on tethering heterochromatin to the nuclear periphery, perhaps it would be relevant to mention that euchromatin-associated factors have recently been proposed to contribute to segregation of heterochromatin, e.g. PMID: 31118512.

Good idea; done.

Page 5, line 24.

The importance of the particular bioinformatics tools used to identify and define LADs is a very relevant aspect. Given the expertise of the Collas group in this aspect, perhaps the authors could provide a figure to illustrate the differences between the cited methods.

We appreciate the comment. It is somewhat challenging to illustrate the principle of the various algorithms used to detect LADs in a didactic way appealing (and understandable) by a broad audience. However, given that (i) the Enriched Domain Detector (EDD) algorithm (which we have published), is quite widely used, and (ii) a critical tunable parameter in EDD (the gap penalty) is easy to illustrate, we propose a figure simply illustrating this important variable in LAD detection (new **Fig. 1d**).

Page 6, lines 48 and 59.

I don't think the citation to ref 61 is correct here. The study by Reddy and coworkers inserted a reporter gene randomly into the genome without knowing (or characterising) if it landed in a LAD or outside.

Citing Ref 61 was indeed inaccurate here; it has been substituted with Guelen 1999 Nature (in ref to line 48) or simply removed (line 59).

Page 7, line 38.

The concept of micro-loops is interesting. Here the authors mention this in the context of ref 71, which is quite recent. However, the concept was proposed much earlier. See for instance Figure 9 in PMID: 21176223 and probably elsewhere.

The reviewer is correct; we now mention that the micro-loop concept was proposed a decade ago {Ikegami, 2010 #3186}.

Page 8, line 51.

Typo: 'nearLAD' -> ' near LAD'

Corrected

Pages 10-11, refs 55 and 59.

I'm a fan of pre-print initiatives like bioRxiv, but I also think that the caution note on the bioRxiv page ('This article is a preprint and has not been certified by peer review') should be taken serious during citation. I haven't looked carefully at the data in refs 55 and 59, nor is it my attention to criticise those manuscripts. However, it strikes me that ref 55 and 59 were posted more that 3 and 2 years ago, respectively, and have not been published in peer-reviewed journals yet. Moreover, comparing the abstracts, a couple of sentences related to the main data of the manuscripts are identical, suggesting that some data from ref 55 were 're-used' again in ref 59. I insist that I'm not saying that the authors should not cite those two pre-preprints, but I suggest them to pay extra attention when citing non-peer-reviewed material.

The reviewer raises a good point. We have reviewed bioRxiv refs 55 and 59 and concluded that due to much redundancy, only Ref 59, which supersedes 55, should be kept. Ref 55 is now removed altogether.

Page 11, line 15.

The observation that LADs relocate to the nuclear periphery was made in ref 58 - and indeed the figure is from this ref - so would be more appropriate to cite ref 58 instead of ref 59.

Indeed; done.

Page 12, line 20.

'Variable chromatin composition and gene expression states lamin A/C-interacting domains suggests…'. I don't understand this sentence. Please rephrase.

Done.

Page 14, line 40.

Please clarify that although the particular cell line used in ref 122 only expresses Dm0, Drosophila has indeed two lamin genes, Dm0 and C.

Done.

Page 16, line 43.

'These diseases…' Please consider adding a reference to this sentence.

Done.

Figures:

-	It seems that Figures 4 and 5 were uploaded in the wrong order. Corrected.

-	Figure 1 legend: 'LAD data (dermal fibroblasts) are from [84].' should be 'ChIP data (dermal fibroblasts) are from [84].' Changed.

-	Figure 1 legend: 'lamin B1using' should be 'lamin B1 using'. Corrected.

-	Figure 3d: What is the purpose of this panel? The information is already present in panel b. Good point; panel removed.

-	Figure 5: Is panel 5b needed? Same phenotype is depicted (and more clearly demonstrated) in 5a. Good point; panel removed.

See revised **Figures 1, 3, 5**.

**Reviewer #2:**

Authors Briand and Collas have provided a timely and thorough review of genome organization through the lens of lamina-associated domains (LADs) and lamin biology. This review describes our current understanding of the role of lamins in positioning lamina-associated domains (LADs) to the nuclear lamina. Overall this review is very timely and provides a needed integration of what is known about lamin biology and LAD organization. I hope that my critiques, summarized below, help to strengthen this very nice review.

Indeed; we much appreciate the insights the reviewer provides. All points are addressed.

Critiques (in order of presentation, not importance)

1) In the abstract there is a sentence that reads "We discuss findings of lamin interactions with regulatory elements of active genes, challenging the dogma of lamins playing a repressive role on transcription." Indeed, in the manuscript the authors offer a much more nuanced view than this sentence would indicate. The majority of data presented in the review supports a major role for LADs being repressive environments, but with some exceptions to that "rule". The sentence in the abstract should reflect this. Please remember this is for a general audience. Something like "While LADs contain mostly repressed genes, we discuss findings of lamin interactions with regulatory elements of active genes, thus expanding our view of the role that lamins may play in genome regulation"

We agree with the nuance here and have rephrased the abstract accordingly, along the lines of what is suggested.

2) The use of the word "anchoring" to describe lamin/LAD interactions is a bit of an issue with me. This suggests a much more solid type of interactions than has been shown by existing data. connotatively this implies a direct and very stable set of interactions. Perhaps better words in lieu of "anchor": associate/association, position/positioning, recruit/recruiting etc.

This could be argued but we see the point and have made changes throughout, using “sequestering”, “association with” or “tethering”.

e.g. in the intro: …a radial disposition enabled by sequestering specific chromosomal domains to the nuclear envelope."

Rephrased.

3)in the paragraph beginning "LADs emerge as…" Should be restated: "LADs emerge as genomic elements that are both shaped by and shape epigenomic states and higher-order genome architecture". LADs are not only driving epigenetic and genome organizational changes, they are also shaped by these changes. You provide the support for this altered statement throughout the manuscript.

Absolutely. Rephrased.

4)"A-type lamins are further processed C-terminally, untethering them…" Incorrect. Only lamin A is processed in this way, lamin C is never farnesylated.

Corrected.

5)The paragraph beginning "the biochemical properties…" are a bit duplicative. These paragraphs should be combined to provide a more fluid narrative.

Agreed: this and the previous paragraph are now combined and redundancy removed (p3).

6)In the paragraph describing super-resolution studies, it is not mentioned that lamin C also forms a distinct network This is important, especially since you mention the preferential filament interactions at the end of this paragraph. I may have missed it,

We did mention that A and C form distinct networks (p3) but now make it more explicit.

…but the microdomains discussed here are also differentially associated with different chromatin and NE components. E.g. lamin C preferentially interacts with NPC (Burke group-proteomics and super-res),

Now mentioned on p3: “Lamins and A and C also differentially associated with NPC subunits [ref Burke].

….while other filaments interact with different INM/chromatin (eg. Shimi et al, and Burke group). I think there is value to describing that here potentially (or in the mediators section)

We have now added this additional point, p3 [ref Shimi 2008, 2015].

7) Zullo et al. also found HDAC3 interacting with Lap2b—this in the context of LAD organization, might be worth mentioning here (this study is mentioned later in the manuscript)

Ref now added in this context (p4).

8) "..a gene repression gradient" Is it a gradient? Or do these associations create a "zone" as described inYao et al 2011 (Tijan group) and suggested in a review by Luperchio et al., 2014. Perhaps this is splitting hairs, but a gradient implies throughout the nucleoplasm, whereas a zone implies a delimited gradient.

Agreed; we have replaced ‘gradient’ with ‘zones’ and cited the appropriate reference.

9)In the paragraph that begins "Accordingly, LADs are rich in .." the Lap2b with HDAC3 should also be mentioned here (can refer back to original description earlier in text).

Done.

10) In the paragraph that begins "On the other hand, this relationship.." the authors describe a set of circumstances in which changes in LADs do not correlate with changes in expression.

a) uncoupling between radial positioning and gene expression level (as stated before, the radial positioning argument falls flat for many reasons

b) genes are "bound to or released from A- or B-type lamins"—the word "bound" is too strong. "interact" is more appropriate.

Rephrased.

c) this raises the separate question of what defines a LAD. If a heterochromatic region is sequestered to the lamina, is being at the lamina necessary for maintaining its heterochromatin identity? In the short term, the answer appears to be "no". Over the longer term, this *may not* be true—e.g. as the Berger group has shown, over time there is a shift in LAD organization in senescence models where lamin B1 is depleted. This reflects perhaps a more nuanced role for sequestration: helping maintain epigenetic state over time.

In short, I think that adding this nuance will help this section and will contextualize these findings a bit more.

We thank the reviewer for this interesting speculation (and suggestion to include this view in this section. We have now done so, p 8.

11) In the paragraph that begins "What are the consequences in changes in LAD identity?" This is confusing. Please change to "What are the consequences in changes in A versus B LAD identity?"

Done

Also, I generally find this nomenclature a bit confusing since A/B compartments are used in HiC data.

Arguably yes; we have rephrased A and B LADs to lamin A and lamin B LADs. We have also removed redundancy in the text in this section.

12) In the section screening for factors mediating locus targeting: I think it is important to discuss several papers that have implicated chromatin state as important for localization: Kind et al, Harr et al., Bian et al., Towbin et al.. This is needed nuance here and can follow your sentence about context dependency.

We now briefly mention these points; note also that we have merged the two subsections “Targeting of loci…” and “ Screening…”, p11.

13) LADs as hotspots for mutations: Since LADs and the B-compartment overlap significantly, I think it is worthwhile to discuss that mechanical forces generated by transiting a micropore seemed to effect B-compartment chromatin specifically (Jacobson, BMC 2018). This also supports a later point in the manuscript musing on the role of mechanotransduction in LAD organization and regulation.

This is a good point and we have added a note on this p16.

14) PTMs: INM proteins are also heavily modified. This should be mentioned and referenced in this

Indeed. Done.
